# Rivaroxaban use during vitamin K_1_ therapy for rodenticide poisoning

**DOI:** 10.1016/j.rpth.2025.103273

**Published:** 2025-11-19

**Authors:** Jean Escal, Angélique Drague, Géraldine Poenou, Pauline Noyel, Laurent Bertoletti, Brigitte Tardy, Xavier Delavenne

**Affiliations:** 1 Laboratoire de pharmacologie et toxicologie, Centre Hospitalier Universitaire de Saint-Etienne, France; 2 Unité Médicale de Recherche 1059 SAnté Ingénierie BIOlogie Saint-Etienne (SAINBIOSE), Institut National de la Santé et de la Recherche Mé dicale, Université de Lyon, Saint-Etienne, France; 3 Service de médecine thérapeutique et vasculaire, Centre Hospitalier Universitaire de Saint-Etienne, France; 4 Laboratoire d’hématologie, Centre Hospitalier Universitaire de Saint-Etienne, France; 5 Centre de Ressources et de Compétence Maladies hémorragiques, Centre Hospitalier Universitaire de Saint-Etienne, France; 6 Centre d'Investigation Clinique 1048, Institut National de la Santé Et de la Recherche Médicale, Centre Hospitalier Universitaire de Saint-Etienne, France

**Keywords:** brodifacoum, case report, difenacoum, difethialone, poisoning, rivaroxaban, rodenticides

## Abstract

**Background:**

Management of severe anticoagulant rodenticide poisoning relies mainly on vitamin K_1_ (VK_1_) therapy, which often requires prolonged administration when long-acting anticoagulant rodenticides (LAARs) are involved.

**Key Clinical Question:**

Can a direct oral anticoagulant (DOAC) be safely reintroduced in a patient at high risk of thrombosis recurrence whose LAAR poisoning is being managed with prolonged VK_1_ therapy?

**Clinical Approach:**

We report the case of a patient receiving rivaroxaban for recurrent thrombosis who ingested massive amounts of brodifacoum, difenacoum, and difethialone over the course of 1 month. He developed severe VK-dependent coagulopathy (international normalized ratio, >26) with hematuria, necessitating discontinuation of rivaroxaban and initiation of intravenous, followed by long-term oral, VK_1_ therapy. Rivaroxaban was successfully reintroduced under close clinical and laboratory monitoring after several days of ongoing oral VK_1_ therapy.

**Conclusion:**

This case demonstrates the feasibility of resuming DOAC therapy during VK_1_ treatment after LAAR poisoning.

## Essentials

• Management of LAAR poisoning requires long-term VK_1_.
• A patient receiving rivaroxaban ingested approximately 1 kg of 2 LAARs.
• Rivaroxaban was successfully reintroduced during long-term VK_1_ therapy with strict monitoring.
• Rivaroxaban therapy can be cautiously resumed during VK_1_ treatment after rodenticides poisoning.

## 1 Introduction

Poisoning with vitamin (V)K antagonist rodenticides occurs frequently in humans worldwide, as these compounds are widely used and readily accessible to the public [1,2]. The management of severe poisoning by these agents relies primarily on the administration of vitamin K_1_ (VK_1_) to control bleeding risk [3]. This risk is particularly pronounced with second-generation anticoagulant rodenticides, known as long-acting anticoagulant rodenticides (LAARs), which possess prolonged biological half-lives [4]. Therefore, VK_1_ therapy often needs to be maintained for several weeks or months following LAAR poisoning [5].

We present a unique case of massive multi-LAAR poisoning in a patient receiving chronic rivaroxaban therapy for recurrent thrombosis, resulting in severe VK-dependent coagulopathy. Long-term management included the reinitiation of rivaroxaban during ongoing oral VK_1_ therapy.

## 2 Case Report

### 2.1 Medical history

A 56-year-old man presented to the emergency department of the University Hospital of Saint-Etienne, France, in February 2025 with macroscopic hematuria associated with lower backpain and asthenia for 2 days. His medical history included multiple thrombotic events between 2014 and 2022, including 2 episodes of pulmonary embolism (PE). He was receiving long-term anticoagulation with rivaroxaban 10 mg daily for secondary prevention of thrombosis. The patient also had a chronic severe anxiety–depressive disorder treated with amitriptyline 100 mg daily.

### 2.2 Initial management and stabilization

On admission, clinical examination revealed only a dry cough with slight diffuse wheezing at the lung bases in addition to the symptoms reported by the patient. Abdominopelvic computed tomography angiography revealed diffuse inflammation of the urinary collecting system and marked bilateral periureteral edema.

Urine cytology showed a markedly elevated red blood cell count (6.77 × 10^6^/mL), while plasma analyses indicated normal renal function (estimated glomerular filtration rate, 85 mL/min/1.73 m^2^), with normal levels of urea, creatinine, glucose, total protein, electrolytes, and hepatic enzymes. Platelet and white blood cell counts were also within normal limits. In contrast, hemoglobin was decreased (112 g/L), and C-reactive protein was elevated (89.4 mg/L). Additionally, the coagulation profile was markedly abnormal: prothrombin time was markedly prolonged, corresponding to <15% activity (normal range, 70%-150%) with an international normalized ratio (INR) exceeding 26 (Figure A), activated partial thromboplastin time ratio was prolonged to 3.27, and fibrinogen was increased to 6.9 g/L. Factor (F)V activity remained normal (81%), ruling out hepatic dysfunction. The plasma rivaroxaban concentration of 39 ng/mL was consistent with therapeutic levels. Consequently, no specific reversal agent was administered, and rivaroxaban therapy was simply suspended. Initial management also included urinary catheterization, repeated bladder irrigations, and the administration of 10 mg intravenous (i.v.) VK_1_ along with analgesics. On the day following hospital admission, the patient was transferred to the urology ward, where bladder irrigations were continued for several days. The urine cleared rapidly, and the patient became pain free.

**Figure:**
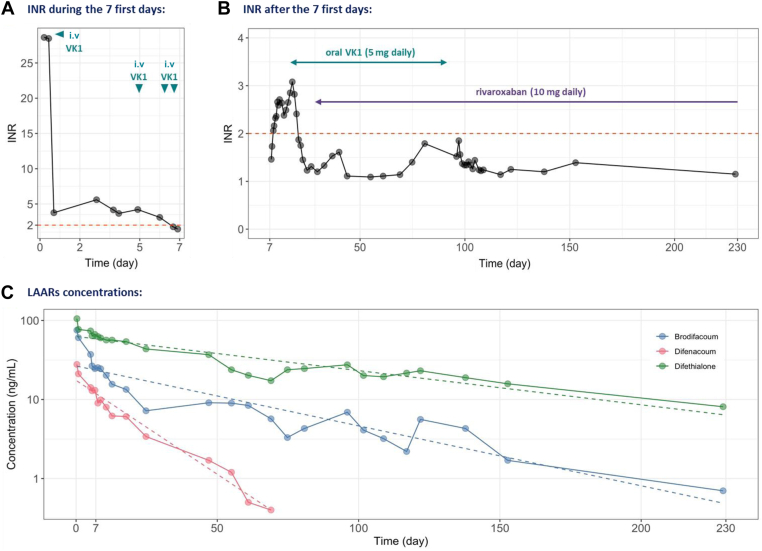
Time course of (A) international normalized ratio (INR) values during the first 7 days of management; (B) INR values after the first 7 days of management; and (C) blood concentrations of long-acting anticoagulant rodenticides (LAARs). The figure indicates when intravenous (i.v.) vitamin (V)K1 or oral vitamin K_1_ and rivaroxaban were administered. Time 0 corresponds to the patient’s arrival at the hospital emergency department.

### 2.3 Unmasking the cause of coagulation disorder

Within a few days, the rivaroxaban plasma concentration became undetectable, and the activated partial thromboplastin time ratio normalized. Conversely, the INR remained elevated (Figure A). This prompted the measurement of VK-dependent coagulation factor activities to further investigate the origin of the coagulation disorder: FII activity was 11%; FVII, 6%; and FX, 8%.

Based on these findings, 2 actions were undertaken: first, readministration of 10 mg i.v. VK_1_, and second, screening and quantification of rodenticides available on the French market. This retrospective toxicological analysis of a whole-blood sample collected on the day of hospital admission revealed 3 LAARs at high concentrations: difethialone, 105.4 ng/mL; brodifacoum, 75.4 ng/mL; and difenacoum, 27.8 ng/mL (Figure C). Repeated toxicological analyses performed on subsequent whole-blood samples over several days demonstrated the prolonged half-lives of all 3 LAARs. Six days after hospital admission, plasma concentrations remained elevated: difethialone, 63.9 ng/mL; brodifacoum, 26.6 ng/mL; and difenacoum, 12.9 ng/mL. During discussion with the medical team, the patient admitted having ingested approximately 1 kg of the 3 rodenticides over the course of 1 month in an attempt to end his psychological suffering. His choice of agents was influenced by the prior suicide of a close relative who had used the same products.

Following this disclosure, treatment consisted of 2 successive 25 mg i.v. VK_1_ infusions >8 hours. This regimen resulted in a normalized INR. However, the corrective effect of VK_1_ was short-lived and the INR increased again within a few days (1.44-3.08) (Figure B). This deterioration prompted the initiation of a maintenance regimen of oral VK_1_ at 5 mg daily. Under this management, the patient remained hemodynamically stable, with a progressive decrease in the INR.

### 2.4 Reintroduction of anticoagulant therapy

After 7 days of 5 mg oral VK_1_ daily, the INR stabilized and remained <2 (Figure B). Consequently, rivaroxaban 10 mg daily was reintroduced, given the patient’s history of multiple thrombotic events, including PE. Clinical examinations were intensified, and daily laboratory monitoring was performed. The patient experienced no recurrence of bleeding, with the INR maintained <2.

### 2.5 From hospital to home: long-term management and VK_1_ withdrawal

After 3 days of combined rivaroxaban 10 mg and 5 mg oral VK_1_ daily, the patient remained clinically stable and biologically normalized. As he had also shown psychological improvement, he was discharged with a daily regimen including 5 mg oral VK_1_, 10 mg rivaroxaban, and 150 mg amitriptyline.

Postdischarge follow-up (clinical evaluation, INR assessment, and measurement of plasma LAARs) was maintained twice weekly for 3 weeks. During one of these follow-ups, imaging of the urinary tract demonstrated normal anatomy and no residual abnormalities.

After 3 weeks, as the patient maintained a stable INR, the follow-up frequency was reduced to once weekly for 2 months. Difenacoum was the first LAAR to become undetectable. As daily measurements were not available, its elimination time was estimated to be approximately 65 days after hospitalization and subsequent discontinuation of exposure. This estimation was based on a calculated half-life (t_1/2_) of 13 days, derived from an estimated elimination rate constant (*k*_e_) of 0.05/d.

Follow-up visits were then spaced every 2 months. During this period, the patient developed a suspected allergic reaction to oral VK_1_. He presented to the emergency department with an episode suggestive of angioedema shortly after taking VK_1_, associated with dyspnea that was partially relieved by inhaled salbutamol. Over the preceding days, he had also experienced facial erythema, painful inflammation of the oral mucosa, dysgeusia, and dysphagia. Clinical examination revealed diffuse erosive lesions of the oral cavity, erythematous and fissured lips, and residual facial erythema. Pruritic plaques were also noted on the hands. The patient received dexchlorpheniramine, and VK_1_ was discontinued (Figure B), with daily INR monitoring and close clinical follow-up.

During subsequent hospitalization, a re-challenge with the usual oral VK_1_ preparation induced no allergic symptoms. Further allergologic investigations identified a drug-induced reaction to proton pump inhibitors that the patient had been dissolving in his mouth instead of swallowing. Despite this clarification, VK_1_ was not reintroduced, as the INR remained consistently <2 and the patient stayed asymptomatic. Brodifacoum elimination then occurred approximately 200 days after stopping the poisoning exposure (calculated t_1/2_, 44 days; estimated *k*_e_, 0.016/d). Difethialone is expected to become undetectable by April 2026, approximately 370 days after exposure ended (calculated t_1/2_, 74 days; estimated *k*_e_, 0.0093/d). Clinical and laboratory monitoring will continue until complete elimination.

Written informed consent was obtained from the patient for publication of this case and accompanying data.

## 3 Discussion

We present the management for an unusual intentional massive ingestion of LAARs in a patient receiving long-term rivaroxaban therapy. To our knowledge, this is the first report describing the successful reintroduction of a direct oral anticoagulant (DOAC) in a patient who was concurrently receiving long-term oral VK_1_ therapy.

Reintroduction of rivaroxaban was primarily justified by the patient’s significant history of recurrent thrombosis, including PE, and by the progressive decline in bleeding risk as the VK_1_ therapy corrected the LAAR-induced coagulopathy. Before reintroducing rivaroxaban, the potential for pharmacodynamic and/or pharmacokinetic interactions between VK_1_ and rivaroxaban was carefully evaluated. This risk was considered minimal, notably because rivaroxaban is a direct FXa inhibitor and therefore acts independently of the VK cycle [6]. Consistent with this, (1) no such interactions have been reported in reviews of DOAC drug–drug interactions [7]; (2) there are no dietary restrictions regarding VK-rich foods for patients treated with DOACs [8]; and (3) current guidelines and reviews on DOAC reversal do not mention VK_1_ or explicitly state that it is ineffective [9,10]. Based on this reasoning, rivaroxaban was cautiously reintroduced once the INR had stabilized <2 and the prothrombin time exceeded 65%. No recurrence of bleeding was observed, suggesting that rivaroxaban therapy can be safely reintroduced during ongoing VK_1_ therapy under careful clinical and laboratory monitoring.

Another main challenge in managing this patient was organizing long-term follow-up over several months. It was important to find a balance between monitoring for possible complications and avoiding excessive medical visits that could disrupt the patient’s daily life. Long-term follow-up and treatment are common in LAAR poisoning, with an average VK_1_ therapy duration of approximately 168 days [4]. In our case, oral VK_1_ treatment lasted 78 days due to a suspected allergic reaction; otherwise, it would have continued longer. The long duration of treatment and follow-up is related to the very slow elimination of LAARs, which mainly results from their accumulation in the liver [11]. In our case, the estimated plasma half-lives were 13 days for difenacoum and 44 days for brodifacoum, consistent with previously reported data [4]. To our knowledge, no pharmacokinetic data are available for difethialone in humans. Its estimated half-life of 72 days is approximately 1.6 times longer than that of brodifacoum, which is already known for its very slow elimination, and approximately 44 times longer than that of warfarin (36-42 hours), which is considered the reference compound among first-generation anticoagulant rodenticides [12].

The predicted disappearance of difethialone from plasma in May 2026 provided crucial information for determining the appropriate duration of patient follow-up. This prediction was made possible through serial measurements of rodenticide plasma concentrations. Such analyses are recommended in current guidelines for the management of rodenticide poisoning [13]. However, a recent review of rodenticide poisoning cases reported that only 43% included confirmed toxicological identification [5]. These observations underline the importance of ensuring that clinical laboratories have validated analytical methods for the detection of all rodenticides, including less commonly encountered compounds [14].
